# Characterization and Therapeutic Potential of Three Depolymerases Against K54 Capsular-Type *Klebsiella pneumoniae*

**DOI:** 10.3390/microorganisms13071544

**Published:** 2025-06-30

**Authors:** Yanjun Lu, Chengju Fang, Li Xiang, Ming Yin, Lvxin Qian, Yi Yan, Luhua Zhang, Ying Li

**Affiliations:** The School of Basic Medical Sciences, Southwest Medical University, Luzhou 646000, China; l710884938@163.com (Y.L.); 18587352508@163.com (C.F.); xiangli20140314@163.com (L.X.); yinming02200059@163.com (M.Y.); m18187115383@163.com (L.Q.); yiyi020127@163.com (Y.Y.)

**Keywords:** carbapenem-resistant hypervirulent *Klebsiella pneumoniae*, phage depolymerases, capsule, serotype K54

## Abstract

Carbapenem-resistant hypervirulent *Klebsiella pneumoniae* (CR-hvKp), a pathogen causing severe nosocomial infections and high mortality rates, is increasingly becoming a serious global public health threat. Capsular polysaccharide (CPS), a major virulence factor of hvKp, can be enzymatically degraded by bacteriophage-derived depolymerases. However, to our knowledge, depolymerases targeting *K. pneumoniae* K54-type strains have rarely been identified. Here, we identified and characterized three novel capsule depolymerases, Dep_C, Dep_Y, and Dep_Z, derived from three different *K. pneumoniae* phages, which retained robust activity across a broad pH range (pH 3.0–12.0) and demonstrated thermal stability up to 50 °C. These depolymerases could efficiently digest the CPS of *K. pneumoniae* K54-serotype strains, significantly inhibit biofilm formation, and remove their mature biofilms. Although no bactericidal activity was detected, these depolymerases rendered host bacteria susceptible to serum complement-mediated killing. We further demonstrate that Dep_C, Dep_Y, and Dep_Z can effectively and significantly prolong the survival time of mice in a pneumonia model infected with K54-type *K. pneumoniae* and reduce the colonization and virulence of the bacteria in the mice. These findings indicate that depolymerases Dep_C, Dep_Y, and Dep_Z could increase bacterial susceptibility to host immune responses of hvKp to the host through their degradation effect on the CPS. In conclusion, our study demonstrates that the three capsule depolymerases are promising antivirulent agents to combat CR-hvKp infections.

## 1. Introduction

*Klebsiella pneumoniae* is a capsulated, facultative anaerobic, and rod-shaped Gram-negative bacterium that resides ubiquitously in the environment [1]. It is an important opportunistic pathogen that can colonize human mucosal surfaces, including the gastrointestinal tract and oropharynx, and disseminate to other tissues, causing several life-threatening infections, including pneumonia, urinary tract infections, bloodstream infections, meningitis, and liver abscesses [1,2]. Classic *K. pneumoniae* commonly causes infections in immunocompromised patients. In contrast, over the past two decades, “hypervirulent” *K. pneumoniae* (hvKp) strains have attracted worldwide attention as they can cause severe and invasive infections (mainly community-acquired) in healthy individuals of any age, with the mortality rate of hvKp infections ranging from 3 to 42% [3]. For a long time, hvKp clinical isolates have remained antibiotic-sensitive. However, more and more multidrug-resistant (MDR) hvKp isolates have emerged in recent years in China, and some exhibit resistance to the last resort antibiotic carbapenems [4,5]. The increasing incidence of carbapenem-resistant hypervirulent *K. pneumoniae* (CR-hvKp) strains poses a serious threat to public health due to the lack of appropriate therapeutic options.

The capsular polysaccharide (CPS) of *K. pneumoniae* is a complex acidic polysaccharide composed of tens to thousands of repeating units of 3–6 monosaccharides [6]. *K. pneumoniae* produces a wide variety of capsule structures, with more than 130 capsular serotypes (K types) identified [6]. The type of capsule appears to correlate with bacterial virulence. At least eight K types, namely, K1, K2, K5, K16, K20, K54, K57, and KN1, have been linked to the hypervirulence of *K. pneumoniae* [7]. The capsule is a critical pathogenic factor for *K. pneumoniae*, conferring resistance to host immune defenses, including phagocytosis and the bactericidal effects of serum [7]. A distinguishing characteristic of the pathogenicity of hvKp strains is their hypermucoviscous phenotype, which is marked by increased amounts of the CPS. Therefore, enzymes with capsule-digesting activity offer antimicrobial potential for combating *K. pneumoniae* infections.

The bacteriophage-encoded depolymerase, a polysaccharide-degrading enzyme present in the phage virion within tail fibers or tail spikes on the baseplate, is capable of targeting and destroying the CPS of bacteria [8]. According to their modes of action, depolymerases can be classified into two main classes, hydrolases and lyases, both leading to the cleavage of polysaccharides into soluble oligosaccharides and the breakdown of the carbohydrate barrier [8,9]. Due to this, depolymerases have been suggested as potential antivirulence agents for fighting bacteria coated with polysaccharide-based capsules [9]. Previous works have demonstrated the therapeutic potential of capsular depolymerases by treating mice infected with *Escherichia coli* [10], *Acinetobacter baumannii* [11], and *K. pneumoniae* [12]. For hvKp strains, phage-encoded depolymerases specific for K1, K2, K5, K20, and K57 types have previously been characterized [13,14]. However, to our knowledge, depolymerases targeting *K. pneumoniae* K54-type strains have rarely been identified. Previously, we reported on the isolation and genome characterization of three phages that infect a clinical *K. pneumoniae* strain of K54 type and can produce polysaccharide depolymerases [15]. Here, we characterized these three K54-specific depolymerases and investigated their therapeutic potential in the treatment of hvKp infections.

## 2. Materials and Methods

### 2.1. Bacterial Strains, Bacteriophages, and Culture Conditions

The bacterial strains, bacteriophages, and plasmids used in this study are listed in Table 1. *K. pneumoniae* strain SCNJ1 is an ST29-K54-serotype CR-hvKp that was recovered from the sputum of a patient in Sichuan Province in November 2018 [16]. The *siphovirus* vB_KpnA_SCNJ1-C (for brevity, referred to here as SCNJ1-C), *myovirus* vB_KpnS_SCNJ1-Y (referred to here as SCNJ1-Y), and *podovirus* vB_KpnM_SCNJ1-Z (referred to here as SCNJ1-Z) were all isolated on the strain SCNJ1 from the sewage water [15]. Unless specified otherwise, all the test strains were cultured in Luria-Bertani (LB) broth with 220 rpm shaking or on LB agar plates at 37 °C. Phages were grown at 37 °C using the host strain SCNJ1. Then, they were collected by centrifugation at 12,000 rpm for 10 min and purified by 0.22 μm filtration (JETBIOFIL, Guangzhou, China).

### 2.2. Bioinformatics Analysis and Evolutionary Tree Construction

MEGA11 [18] was used to construct a phylogenetic tree of the putative proteins with homology from BLASTp comparison. Amino acid sequences of depolymerases encoded by other *K. pneumoniae* bacteriophages were retrieved from GenBank. The amino acid sequences of the three depolymerases were aligned with each other by Clustal X (1.81) [19] and visualized by ESPript3.0 [20]. The secondary structure of depolymerases was predicted by SWISS-MODEL [21]. The conserved domains of three depolymerases were analyzed by InterPro [22] and SWISS-MODEL, InterPro, and BATCH CD-search are all webpages rather than software, so there is no version information. [23].

### 2.3. Cloning of Putative CPS Depolymerase Genes and Protein Expression

Target genes were amplified via PCR with phage genomic DNA as templates usin g the primers: Dep_Z(F): 5′-tatcggaattaattcggatccGATGGCATTCAGCTGGCAA-3′, Dep_Z(R): 5′-gtggtggtggtggtgctcgagTACTACTGTGCCAGTTGAGTCCACC-3′, Dep_C(F): 5′-tatcggaattaattcggatccGATGGCTACTACCCCAACTAACAAG-3′, and Dep_C(R): 5′-gtggtggtggtggtgctcgagAACAGCTGTGCCAGTAGCATCTAC-3′. The resulting PCR products were inserted into the BamHI/XhoI-digested pET22b(+) plasmid using the ClonExpress II One Step Cloning Kit (Vazyme, Nanjing, China). The resulting recombinant plasmids were transformed into *E. coli* BL21(DE3) cells for protein expression.

The expression of Dep_C or Dep_Z was induced with 0.5 mM of isopropyl-β-D-thiogalactopyranoside (IPTG) [24] and incubated at 25 °C for 12 h. Cells were harvested by centrifugation (at 8000 rpm and 4 °C for 10 min) and resuspended in ice-cold phosphate-buffered saline (PBS, pH 7.4). Cell lysis was performed using an ultrasonic disruptor (200 W output) in an ice bath with 5 s pulse/3 s interval cycles for a 30 min total duration. The lysate was clarified by centrifugation (at 12,000 rpm and 4 °C for 20 min) and filtered through a 0.22 μm filter. Proteins were purified using a 1 mL HisTrap^TM^ Ni-NTA column (Cytiva, Marlborough, MA, USA) according to the manufacturer’s instructions. The purified recombinant protein was eluted at a concentration of 100 mM of imidazole and analyzed using 12% gradient sodium dodecyl sulfate–polyacrylamide gel electrophoresis (12% SDS-PAGE). The purified single-band solution was concentrated using Merck Millipore ultrafiltration centrifuge tubes. The concentration of recombinant protein was determined using the Bradford method [25]. The purified protein was stored at −80 °C.

### 2.4. pH and Thermal Stability Analyses

The stability of depolymerases was evaluated via a plaque assay under different pH (pH 3.0–12.0) and thermal (20–60 °C) conditions [26]. Next, 1 mL of log-phase *K. pneumoniae* SCNJ1 cultures [the optical density at 600 nm (OD600 nm)~0.5] were mixed with 1 mL of the LB semi-solid medium. Then, the mixture was overlaid on solid LB plates. For temperature stability analysis, the depolymerases were diluted with 100 mM of imidazole buffer. Then, 20 μL of the depolymerases Dep_C (at doses of 40, 30, 20, or 10 ng), Dep_Z (at doses of 40, 30, 20, or 10 ng), or Dep_Y (at doses of 120, 60, 24, or 12 ng) was incubated at 20 °C, 30 °C, 40 °C, 50 °C, or 60 °C for 1 h. For pH stability analysis, the pH of 100 mM of imidazole was adjusted to a range of 3 to 12 with 1 M of NaOH or 1 M of HCl. Then, the depolymerases were diluted with the pH-adjusted imidazole buffer. Next, 5 μL of Dep_C (at a dose of 10, 7.5, 5, or 2.5 ng), Dep_Z (at a dose of 10, 7.5, 5, or 2.5 ng), or Dep_Y (at a dose of 30, 15, 6, or 3 ng) was spotted on the plate containing SCNJ1, with 100 mM of imidazole as the negative control. After incubation at 37 °C for 12 h, the appearance of a transparent halo on the LB agar indicated that the depolymerase was active.

### 2.5. Determination of Host Range and Capsule Depolymerase Activity

The activity ranges of capsular depolymerases were determined via a plaque assay [14]. We used 10 different bacterial strains for the host range identification of depolymerases, including *A. baumannii*, *Enterobacter cloacae*, *E. coli*, and *K. pneumoniae*, with different capsular serotypes. An LB agar plate was overlaid with top agar containing a mixture of 1 mL of fresh bacterial culture and 1 mL of LB semi-solid medium. Next, 5 μL of depolymerase (50 ng) was spotted on the plate, with 100 mM of imidazole as the negative control. After incubation at 37 °C for 12 h, the appearance of a transparent halo on the LB agar indicated that the depolymerase was active.

Depolymerase activity was also determined via a plaque assay [26]. First, 1 mL of a log-phase *K. pneumoniae* SCNJ1 culture (OD600 nm~0.5) was mixed with 1 mL of the LB semi-solid medium and overlaid on an LB agar plate. Next, 5 μL of depolymerases (at a dose of 250, 125, 50, 5, or 0.5 ng) was spotted. In the experiment, 100 mM of imidazole served as the negative control. After incubating at 37 °C for 12 h, the appearance of a transparent halo on the LB agar indicated that the depolymerase was active.

### 2.6. Phage Adsorption Inhibition Test

The effect of depolymerases on the adsorption of bacteriophage to *K. pneumoniae* SCNJ1 was evaluated, as previously described with some modifications [13]. The phages (SCNJ1-C, SCNJ1-Y, and SCNJ1-Z) were subjected to the quantitative determination of the base number (10^5^ PFU/mL). First, 100 μL of SCNJ1 (10^8^ CFU/mL) was incubated with 100 μL of depolymerases (10 μg/mL) at 37 °C for 15 min. A sucrose magnesium buffer (SM buffer) served as the negative control. Next, 100 μL of the phages (10^4^ PFU/mL) was added to the mixture. After incubation at 37 °C for 10 min, bacterial cells with the adsorbed phages were precipitated by centrifugation at 12,000 rpm for 10 min. The supernatant containing free phages was mixed with the bacterial culture of SCNJ1 (10^8^ CFU/mL) at a 1:1 *v*/*v* ratio, followed by incubation at 37 °C for 10 min. The suspension and molten LB-top agar were gently mixed (1:1 *v*/*v* ratio) and distributed onto LB agar plates. After solidification, the plates were incubated at 37 °C for 12 h. The non-adsorbed phages were counted and recorded as PFUs. The experiment was repeated at least three times in triplicate.

### 2.7. Extraction of CPS and Alcian Blue Staining

The CPS extraction from *K. pneumoniae* SCNJ1 was performed, as described in [14]. *K. pneumoniae* SCNJ1 was cultured and adjusted to an OD600 nm of 1.0. Bacterial cells were mixed with 1% Zwitterant 3–14 (5:1 *v*/*v* ratio), and citric acid was added to a final concentration of 100 mM, followed by incubation at 50 °C for 45 min with mixing every 15 min. The supernatant was collected after centrifugation at 13,000 rpm for 20 min, and anhydrous ethanol was added to a final concentration of 80%. After that, the mixture was allowed to precipitate overnight at 4 °C. The supernatant was discarded after centrifugation at 13,000 rpm for 20 min, and the capsule precipitate was dissolved in 10 mL of distilled water after drying. Subsequently, the suspension was treated with DNase/RNase and incubated at 37 °C for 2 h, followed by protease K treatment at 55 °C for 2 h. The sample was lyophilized for 24 h and stored at −20 °C. The CPS was dissolved in sterile water before use.

The degradation of the CPS was determined by gel electrophoresis followed by Alcian blue staining. First, 8 μg of the CPS dilution was mixed with 3 μg of depolymerases or an equal volume of ddH_2_O (negative control) at 37 °C for 2 h [27]. An SDS-PAGE loading buffer (Beyotime, Shanghai, China) was added to the samples, which were then boiled for 10 min. Next, an 8% separating gel and a stacking gel were solidified at room temperature, and 10 μL of the sample was added into the loading well, followed by SDS-PAGE electrophoresis. Then, the gel was washed in a fixing buffer (25% ethanol and 10% acetic acid in ddH_2_O) three times at 50 °C for 10 min before staining with 0.125% Alcian blue in a fixing buffer at 50 °C for 15–20 min in the dark. The gel was decolorized with the fixing buffer at room temperature.

### 2.8. Activity Assay

The activity of depolymerases was assessed by measuring the decrease in turbidity, according to the method with some modifications [28]. First, 100 μL of the CPS of *K. pneumoniae* SCNJ1 (SCNJ1-CPS, 300 μg/mL) was mixed with 100 μL of Dep_C, Dep_Y, or Dep_Z (10 μg/mL) and incubated at 37 °C for 2 h. After that, the mixture was treated with cetylpyridinium chloride (CPC) to a final concentration of 5 mg/mL and incubated at room temperature for 5 min. The CPS treated with an equal volume of PBS was used as the negative control. The values of OD600 nm were measured using a microplate reader (BioTek, Winoosk, VT, USA). Each test was performed in at least three independent experiments.

### 2.9. Biofilm Formation Inhibition Assay and Biofilm Degradation Assay

A biofilm formation inhibition assay was determined using crystal violet staining on a 96-well microtiter plate with some modifications [29]. The log-phase *K. pneumoniae* SCNJ1 was adjusted to an OD600 of 0.1 (~2.0 × 10^8^ CFU/mL), diluted 1:100 in the LB medium, and then added to a sterile 96-well microtiter plate (195 μL/well). Next, 5 μL of depolymerases Dep_C, Dep_Y, or Dep_Z (at a dose of 50 ng, 100 ng, or 200 ng) was added to each well. The samples were incubated at 37 °C for 24 h, respectively. The biofilm degradation assay was modified based on a previous method [30]. The log-phase SCNJ1 was adjusted to an OD600 nm of 0.1 (~2.0 × 10^8^ CFU/mL). After being diluted 100-fold with fresh LB, 200 μL of the bacterial culture was transferred to a 96-well flat-bottom microplate and incubated at 37 °C for 48 h. Then, the bacterial culture was removed, and the wells were washed three times with PBS. Finally, 200 μL of depolymerases (at a dose of 50 ng, 100 ng, or 200 ng) or an equal volume of PBS was added to each well and incubated for 6 h at 37 °C.

The amount of biofilm in each well was determined using crystal violet staining. The bacterial culture was removed, and each well was washed three times with PBS and dried. The biofilms in the wells were fixed with methanol for 15 min before being washed twice and dried. After that, the wells were stained with a crystal violet solution (0.1%, *w*/*v*) for 20 min and then washed twice and dried. The bound dye was dissolved in 200 μL of 33% glacial acetic acid for 10 min, and the OD595 nm was measured to determine the quantity of the biofilms. The experiment was repeated at least three times in triplicate.

### 2.10. Serum Killing Assay

The survival rate of *K. pneumoniae* SCNJ1 after pretreatment with depolymerases and subsequent killing by the serum was evaluated [31]. The log-phase *K. pneumoniae* SCNJ1 was adjusted to an OD600nm of 0.1 (~2.0 × 10^8^ CFU/mL). Next, 10^6^ CFU of log-phase SCNJ1 was pretreated with depolymerases at a final concentration of 150 μg/mL in a final volume of 10 μL, followed by incubation at 37 °C for one hour. SCNJ1 with an equal volume of PBS was set up as a control. Then, bacterial cells diluted 10-fold with PBS (10^5^ CFU) were treated with human serum from healthy volunteers at a 1:3 *v*/*v* ratio so that the concentration of serum reached 75% in a final volume of 400 μL. Meanwhile, control groups were set up in which the bacteria were pretreated with depolymerases or PBS, but no serum was added. The mixture was incubated at 37 °C for 3 h, and the viable bacteria were evaluated by CFU counting through serial dilution and plating on LB agar plates. The bacterial survival rates were calculated based on viable counts relative to the initial inoculum. The experiment was repeated at least three times in triplicate.

### 2.11. Mouse Infection Model

Animal experiments were approved by the Southwest Medical University Institutional Animal Care and Use Committee, and we have complied with all relevant ethical regulations for animal use. Female BALB/c mice aged 6–8 weeks were obtained from Jiangsu Huachuang Sino Pharma Tech (Taizhou, China). Before and after vaccination, the mice had unrestricted access to food and water.

A mouse pneumonia model was established, as described in our previous study [32]. To evaluate therapeutic efficacy, mice were randomly divided into 4 experimental groups (12 mice/group) and 1 control group (3 mice/group). After being lightly anesthetized by the inhalation of isoflurane, each mouse in the experimental group was intranasally infected with 50 μL of *K. pneumoniae* SCNJ1 (1 × 10^7^ CFU). Mice in the control group were intranasally inoculated with 50 μL of sterile PBS. One hour after infection, 50 μL of depolymerases (at a dose of 30 μg per mouse) or 50 μL of PBS buffer was administered via intranasal inoculation. In addition, three safety groups were set (5 mice/group). Mice in these groups were administered 50 μL of depolymerases (at a dose of 30 μg per mouse) via intranasal inoculation without SCNJ1 infection. Mice in all groups were monitored daily for 7 days.

To further evaluate the therapeutic effect of depolymerase, we determined the bacterial titer in the lungs, liver, and spleen of the infected mice. Mice were randomized into five groups: depolymerase-treated groups (5 mice/group), a PBS-treated group (5 mice/group), and a PBS control group (3 mice/group). After being lightly anesthetized by the inhalation of isoflurane, mice in the depolymerase- and PBS-treated groups were intranasally infected with 50 μL of SCNJ1 (1 × 10^6^ CFU). One hour later, they were treated with 50 μL of depolymerases (at a dose of 20 μg per mouse) or 50 μL of PBS intranasally. After 48 h, the mice were euthanized, and their lungs, livers, and spleens were dissected. The organs were weighed and homogenized in sterile PBS. The homogenates were serially diluted and plated on LB agar plates for counting the colonies. The CFU count obtained for each organ was normalized based on 1 g of the wet organ weight.

### 2.12. Histopathology

For histologic analysis, after being lightly anesthetized by the inhalation of isoflurane, each mouse was intranasally infected with 50 μL of SCNJ1 (1 × 10^6^ CFU). One hour later, they were treated with 50 μL of depolymerases (at a dose of 20 μg per mouse) or 50 μL of PBS intranasally. As a control, three mice were intranasally inoculated with 50 μL of sterile PBS. After 48 h, the left lungs of the mice were removed, washed in PBS, fixed in 10% neutral buffered formalin, dehydrated in ethanol, and embedded in paraffin. The embedded tissues were sectioned at 5 μm and dried before staining with hematoxylin and eosin. Images were obtained using an Olympus BX43 microscope (Olympus, Tokyo, Japan) and a Sony E3ISPM20000KPA camera (Sony, Tokyo, Japan). Figures were created using ImageView 4.11.22149.

### 2.13. Statistical Analysis 

Experimental data are presented as mean ± SD. Statistical analysis was performed using Prism 10.1.2 (GraphPad Software, La Jolla, CA, USA). One-way ANOVA compared multiple groups for phage adsorption inhibition, depolymerase activity, biofilm formation inhibition, biofilm degradation, and serum killing assays. In the mouse colonization experiment, the Kruskal–Wallis (KW) analysis was employed to assess the bacterial load in the spleen. A two-tailed Mann–Whitney U test was utilized to analyze the bacterial loads in both the lungs and the liver. The survival curves are analyzed by the log-rank (Mantel–Cox) test. * *p* < 0.05, ** *p* < 0.01, *** *p* < 0.001, and **** *p* < 0.0001 were treated as the levels of statistical significance.

## 3. Results

### 3.1. Bioinformatics Analysis of Depolymerase

**Figure 1:**
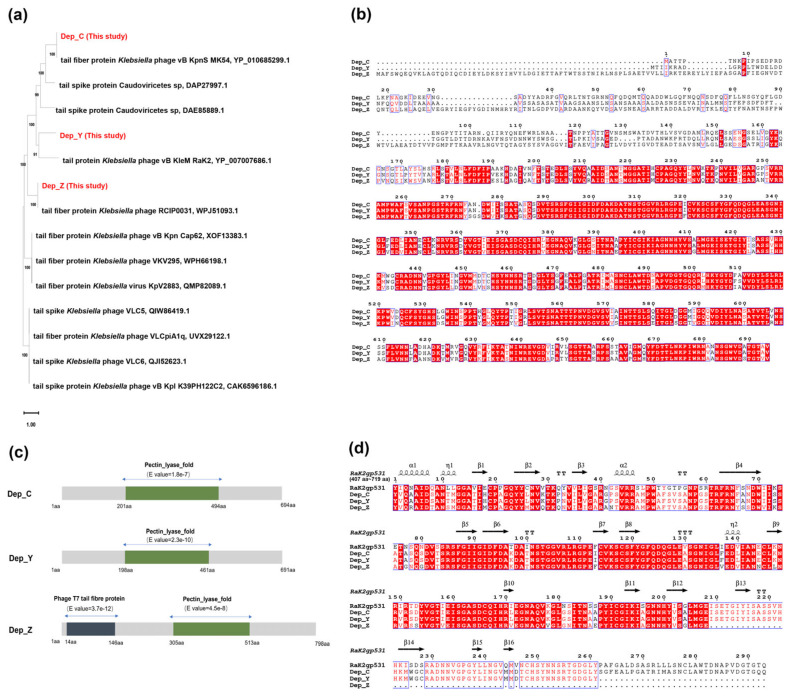
The bioinformatics analysis of depolymerases. (**a**) The phylogenetic analyses of Dep_C, Dep_Y, and Dep_Z. The phylogenetic tree was built using MEGA11 based on amino acid sequences of the tail fiber and tail spike proteins using the maximum likelihood method with 1000 bootstrap replicates. (**b**) The alignment of the full amino acid sequences of Dep_C, Dep_Y, and Dep_Z. The amino acid sequences were compared by Clustal X and visualized by ESPript 3.0. The background of identical residues is marked in red, and the homologous residues are highlighted in red. (**c**) The protein domains were predicted by InterPro and BATCH CD-search. (**d**) The alignment of the amino acid sequences of the pectin_lyase_fold structure of RaK2gp531 (407 aa~719 aa, E value = 4.16 × 10^−6^), Dep_C, Dep_Y, and Dep_Z. The secondary structure of RaK2gp531 is predicted by SWISS-MODEL. In this structure, α-helices are represented by coils, and β-strands are represented by arrows. The background of identical residues is marked in red, and the homologous residues are highlighted in red.

We identified three putative depolymerase-encoding genes, designated as *dep_Z* (orf57 of SCNJ1-Z, accession number: OQ689084), *dep_C* (orf50 of SCNJ1-C, accession number: OQ718882), and *dep_Y* (orf40 of SCNJ1-Y, accession number: OQ689083). They were predicted to be phage tail fiber proteins or phage tail spike proteins. We compared their amino acid sequences with the top 15 matching results among other putative phage tail fiber proteins or tail spike proteins in the GenBank database. The phylogenetic tree shows that Dep_C is on a separate branch with the tail fiber protein of *K. pneumoniae* bacteriophage vB_KpnS_MK54, and Dep_Y is clustered with the phage vB_KleM_RaK2 tail protein gp531, which is effective against the *K. pneumoniae* K54-serotype capsule [33] (Figure 1a). Dep_Z is closely related to the tail fiber of the *K. pneumoniae* bacteriophage RCIP0031. From the phylogenetic tree, Dep_C and Dep_Y are more closely related to each other than Dep_Z is to either of them. The alignment of full amino acid sequences of Dep_C, Dep_Y, and Dep_Z shows that the identity between Dep_Y and Dep_C is 91.9%, while Dep_Z only shows 75.78% and 75.14% to Dep_Y and Dep_Z, respectively (Figure 1b).

We also predicted the protein domains of Dep_C, Dep_Y, and Dep_Z by InterPro and BATCH CD-search. Dep_Z, but not Dep_C and Dep_Y, has a conserved domain (14 aa~146 aa) in the N-terminal region, which shows similarity to the tail fiber protein of phage T7 (E value = 3.7 × 10^−12^). In the central domains of the amino acid coding sequence of Dep_C (201 aa~494 aa, E value = 1.8 × 10^−7^), Dep_Y (198 aa~461 aa, E value = 2.3 × 10^−10^), and Dep_Z (305 aa~513 aa, E value = 4.5 × 10^−8^), there is a stretch of sequence belonging to the pectin lyase homologous superfamily, which is predicted to be Pectin_lyase_fold (Figure 1c).

Before this study, RaK2gp531 remained the sole documented depolymerase exhibiting activity against the K54-serotype CPS of *K. pneumoniae* [33]. In the central region of the amino acid coding sequence of RaK2gp531 (407 aa~719 aa, E value = 4.16 × 10^−6^), a pectin_lyase_fold structure is also present, which shows high similarity (Figure 1d) to those in Dep_C (77.71% identity), Dep_Y (77.71% identity), and Dep_Z (74.92% identity). In addition, the amino acid sequence of the C-terminal of RaK2gp531 shows >70% identity to those of Dep_C, Dep_Y, and Dep_Z, which may be related to their specific recognition of the CPS of the K54-serotype *K. pneumoniae*.

### 3.2. Expression of the Putative Capsule Depolymerases

**Figure 2:**
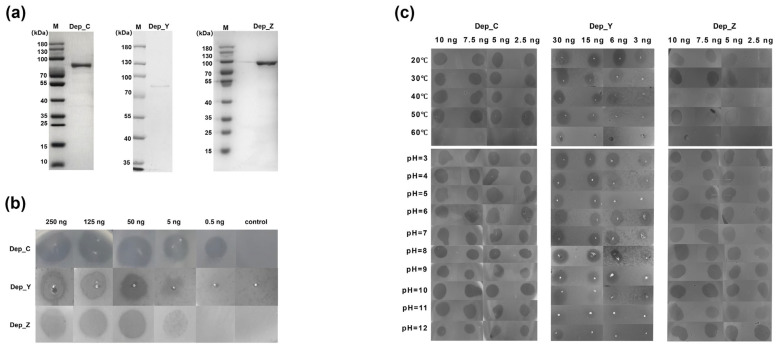
The expression, activity, and stability of the depolymerases. (**a**) The 12% SDS-PAGE electrophoresis of the purified recombinant proteins. M, marker. (**b**) The activity assays of the three depolymerases. The appearance of a halo on the plate indicates that the depolymerase is active. (**c**) The stability of the three depolymerases at different pH (pH 3.0–12.0) and different temperatures (20–60 °C). The appearance of a halo on the plate indicates that the depolymerase is active.

We cloned and expressed Dep_Z and Dep_C in *E. coli* via a pET22b(+) expression system in this work. The recombinant protein Dep_Y was expressed in our previous study (unpublished data). His-tag fusion proteins Dep_Z, Dep_C, and Dep_Y, with a predicted molecular mass of 87.8 kDa, 76.3 kDa, and 73.7 kDa, respectively, were purified by affinity chromatography (Figure 2a). To determine their enzymatic activities, the purified recombinant proteins were assessed by spot tests against the *K. pneumoniae* strain SCNJ1. The generation of translucent zones of clearance on the bacterial lawn manifests their CPS depolymerization activity. When different amounts of depolymerases were tested, translucent halos were formed with ≥5 ng of Dep_Z, while a relatively weak halo was formed with 5 ng of Dep_Y. Dep_C showed the highest activity as its halo zone was detectable at concentrations as low as 0.5 ng (Figure 2b). We also tested their activities on seven *K. pneumoniae* strains representing different K types and three other bacterial species. Dep_Z, Dep_C, and Dep_Y were only active against the K54-type *K. pneumoniae* strain SCNJ9 and not on any of the others (Table 2), confirming their specificity to K54-capsulated *K. pneumoniae*.

An assessment of the pH and thermal stability of Dep_C, Dep_Y, and Dep_Z was further carried out. The results show that both Dep_C and Dep_Z were highly active from pH 3.0 to pH 12.0, with their translucent halos being noticeable at concentrations as low as 2.5 ng (Figure 2c). In contrast, Dep_Y showed relatively lower stability in a changing pH environment, and its enzymatic activity was significantly reduced at pH > 9. In addition, Dep_C and Dep_Y retain stable activity from 20 °C to 50 °C, while Dep_Z was sensitive to temperature change, with clear halos formed only at 30 °C (Figure 2c).

### 3.3. Depolymerases Inhibit the Adsorption of Phages on Bacterial Cells

**Figure 3:**
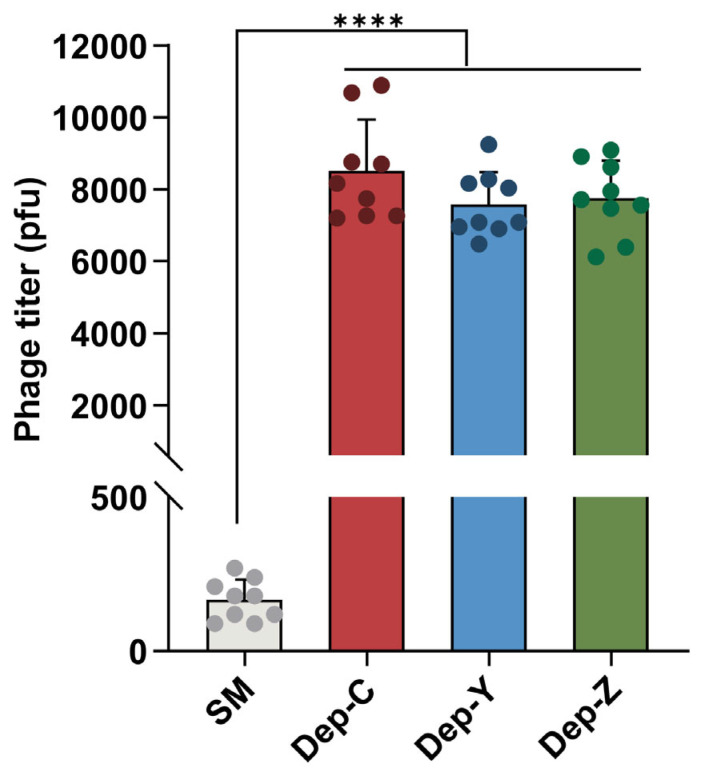
Heterologously expressed depolymerases inhibit the adsorption of phages on bacterial cells. *K. pneumoniae* SCNJ1, pretreated with three different depolymerases, was infected with phages SCNJ1-C, SCNJ1-Y, or SCNJ1-Z. An equal volume of the SM buffer was used as a negative control. Data are presented as the mean ± SD (*n* = 3) from at least three independent experiments (in triplicate). Each dot represents a repeated value, and error bars represent the standard error of the mean. Statistical analyses were performed using one-way ANOVA analysis, **** *p* < 0.0001.

Previous studies demonstrated that the CPS serves as a primary phage receptor and is responsible for the attachment of phages to bacterial cells in *K. pneumoniae* [34]. To determine the contributions of depolymerases in the *K. pneumoniae* SCNJ1 phage–cell initial interaction, we performed phage adsorption inhibition assays. After being pretreated with the depolymerase, the number of free phages in the supernatant after centrifugation increased significantly compared with that in the untreated SCNJ1 (all, *p* < 0.0001, Figure 3). This indicates that the phage adsorption to bacterial cells is inhibited by depolymerase, which may be because viral depolymerases and heterologously expressed depolymerases compete for the CPS on the cell surface.

### 3.4. Determination of Capsule-Digesting Activities of Depolymerases

**Figure 4:**
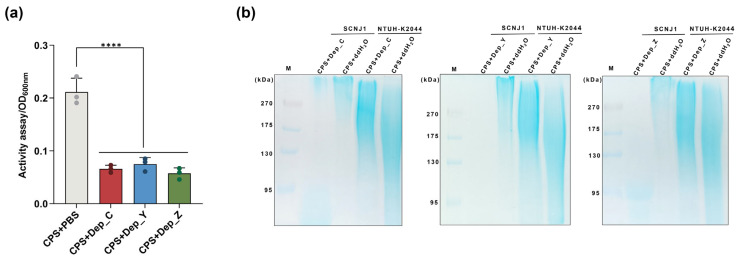
The determination of the capsule-digesting activities of depolymerases. (**a**) Dep_C, Dep_Y, or Dep_Z were incubated with the CPS of *K. pneumoniae* SCNJ1. The CPS of SCNJ1 mixed with PBS was used as the negative control. After adding CPC, the degradation effects of depolymerases on the CPS were measured by OD600 nm. Data are presented as the mean ± SD (*n* = 3) from at least three independent experiments. Each dot represents a repeated value and error bars represent the standard error of the mean. Statistical analyses were performed using one-way ANOVA analysis, **** *p* < 0.0001. (**b**) Alcian blue staining of the CPS of *K. pneumoniae* SCNJ1 treated with Dep_C, Dep_Y, or Dep_Z. The CPS treated with ddH_2_O served as a control. An unrelated strain, NTUH-K2044 (CPS type: K1), was used as a negative control.

To explore their ability to digest CPSs, the degradation of the CPS extracted from *K. pneumoniae* SCNJ1 was evaluated by measuring the decrease in turbidity (the release of reducing sugars was precipitated by CPC) after incubating with depolymerase. As shown in Figure 4a, after incubation with Dep_C, Dep_Y, or Dep_Z for 2 h, the OD600 nm values of CPS were 0.07 ± 0.01, 0.08 ± 0.01, and 0.06 ± 0.01, respectively, significantly lower (all, *p* < 0.0001) than that of incubation with PBS (0.21 ± 0.03). The results demonstrate the enzymatic degradation of CPS by depolymerases.

The capsule-degrading property of depolymerases was further visualized by the Alcian blue staining of depolymerase-treated CPS. The results of the SDS-PAGE gel reveal that the CPS of *K. pneumoniae* SCNJ1 (K54-type) was degraded by Dep_Z, Dep_C, or Dep_Y, respectively. Compared to the PBS-treated CPS, the high molecular weight polysaccharide polymers disappeared, and lower molecular weight material was observed after depolymerase treatment, revealing the capsule degradation activities of these depolymerases (Figure 4b). An irrelevant K1-type *K. pneumoniae* strain NTUH-K2044 was used as a control. The absence of degradation activities for Dep_Z, Dep_C, or Dep_Y against the K1-type CPS confirms their high specificity (Figure 4b).

### 3.5. Depolymerases Are Effective in Inhibiting Biofilm Formation and Degrading Pre-Formed Biofilms of K. pneumoniae

**Figure 5:**
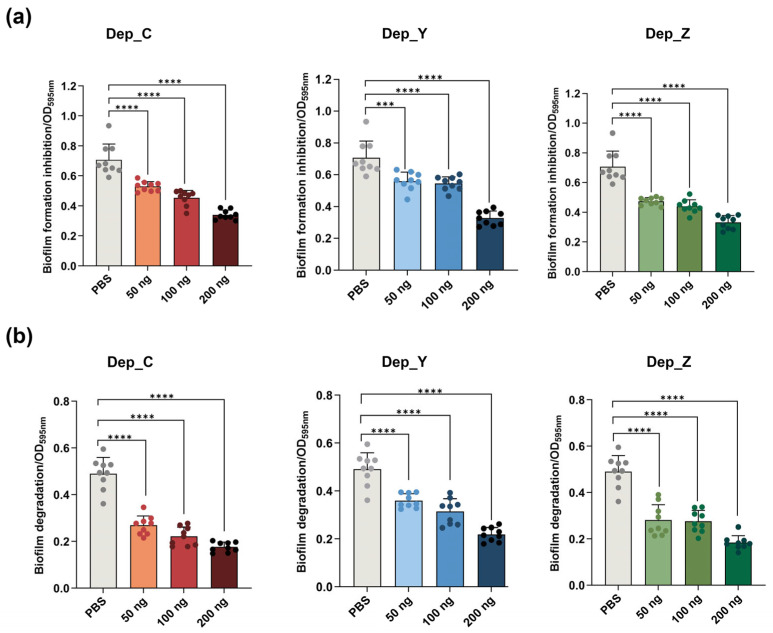
The degradation and inhibitory effects of depolymerases on the *K. pneumoniae* SCNJ1 biofilm. (**a**) Dep_C, Dep_Y, or Dep_Z (50 ng, 100 ng, or 200 ng) was incubated with *K. pneumoniae* SCNJ1 for 24h. SCNJ1 incubated with PBS was used as the control. The inhibitory effects of depolymerases on the biofilm were measured by OD595 nm. Data are presented as the mean ± SD (*n* = 3) from at least three independent experiments (in triplicate). Each dot represents a repeated value and error bars represent the standard error of the mean. Statistical analyses were performed using one-way ANOVA analysis, **** *p* < 0.0001. *** *p* < 0.001. (**b**) Dep_C, Dep_Y, or Dep_Z (50 ng, 100 ng, or 200 ng) were incubated with 48 h mature biofilms of SCNJ1 for 6 h. The SCNJ1 biofilm treated with PBS was used as the control. The degradation effects of depolymerases on the biofilm were measured by OD595 nm. Data are presented as the mean ± SD (*n* = 3) from at least three independent experiments (in triplicate), and error bars represent the standard error of the mean. Statistical analyses were performed using one-way ANOVA analysis, **** *p* < 0.0001.

Bacterial biofilm can prevent the penetration of drugs through its matrix, reducing the ability of antibiotics to reach the surface of bacteria [35]. Extracellular polysaccharides (EPSs) are a major component of the biofilm matrix [36]. To explore the inhibitory effects of depolymerases Dep_C, Dep_Y, and Dep_Z on the biofilm formation of *K. pneumoniae* SCNJ1, depolymerases (at a dose of 50 ng, 100 ng, or 200 ng) were co-cultured with the host bacteria for 24 h. As shown in Figure 5a, compared with the PBS treatment group, the amount of biofilms in the Dep_C, Dep_Y, and Dep_Z treatment groups significantly decreased in a dose-dependent manner (except for the 50 ng Dep_Y, *p* < 0.001; all *p* < 0.0001). The results indicate that depolymerases can effectively inhibit the biofilm formation of *K. pneumoniae* SCNJ1.

To investigate the effect of depolymerases on degrading *K. pneumoniae* biofilms, mature biofilms, grown for 48 h, were exposed to depolymerases, followed by a crystal violet staining assay. As shown in Figure 5b, compared with the group treated with PBS, the amount of biofilms treated with Dep_Z, Dep_C, or Dep_Y (at a dose of 50 ng, 100 ng, or 200 ng) was significantly reduced in a dose-dependent manner (all, *p* < 0.0001). The results indicate that depolymerases are effective in eradicating biofilms formed by *K. pneumoniae* SCNJ1.

### 3.6. Depolymerase-Treated Bacteria Become Sensitive to Serum Killing

**Figure 6:**
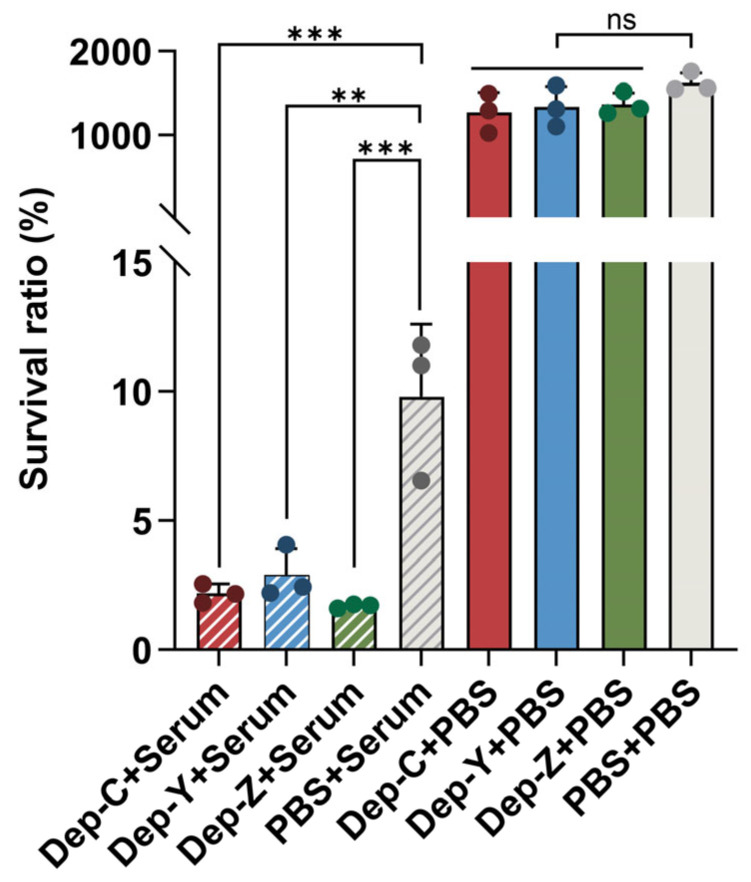
The killing effect of serum on SCNJ1 with the pretreatment of depolymerase. The survival rate is expressed as the percentage of the number of viable bacteria after incubation with serum or PBS for 3 h relative to the viable count of the initial inoculum. Bacteria pretreated with depolymerases or PBS but no serum added serve as control groups. Data are presented as the mean ± SD (*n* = 3) from at least three independent experiments. Each dot represents a repeated value and error bars represent the standard error of the mean. Statistical analyses were performed using one-way ANOVA analysis. ** *p* < 0.01 and *** *p* < 0.001.

It has been known that CPS protects bacteria against complement-mediated killing in serum [37]. Here, the killing effect on the strain SCNJ1 was evaluated after incubation with 75% human serum for 3 h, either with or without the pretreatment of capsular depolymerases. The results show that compared with the group that was pretreated with PBS, bacteria pretreated with depolymerases became sensitive to serum killing, and the bacterial survival rate was significantly decreased (Dep_C, *p* = 0.0008; Dep_Y, *p* = 0.0016; Dep_Z, *p* = 0.0004, Figure 6). Meanwhile, control groups were set up in which the bacteria were pretreated with depolymerase, but no serum was added. In these groups, the bacterial load was comparable to the PBS-treated group, indicating that Dep_C, Dep_Y, or Dep_Z alone had no killing effect on SCNJ1. Therefore, our results collectively suggest that after the capsular depolymerases degrade the capsule, they can enhance the sensitivity of bacteria to serum-mediated killing.

### 3.7. Depolymerase Treatment Rescues Mice Infected with K54 Hypervirulent K. pneumoniae

**Figure 7:**
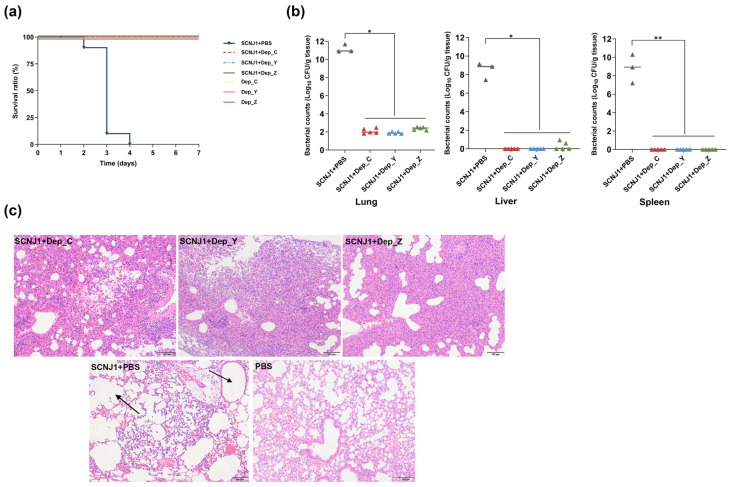
The therapeutic effects of Dep_C, Dep_Y, and Dep_Z in a mouse pneumonia model. (**a**) The survival curves of mice (*n* = 12 for each group) intranasally infected with SCNJ1 (1 × 10^7^ CFU) followed by treatment with depolymerases (30 μg/mouse) or PBS. Mice in the safety groups (*n* = 5 for each group) were only inoculated with depolymerases (30 μg/mouse). The survival state of mice was recorded every 24 h for 7 consecutive days, and statistical analyses were performed using the log-rank (Mantel–Cox) test. (**b**) Bacterial loads in the lungs, liver, and spleen samples of mice. Mice were treated with 50 μL of Dep_C, Dep_Y, or Dep_Z (at a dose of 20 μg per mouse) or 50 μL of PBS one hour after intranasal infection with SCNJ1 (1 × 10^6^ CFU). Samples of the lungs, liver, and spleen were collected from the mice 48 h later, and the bacterial loads were determined by counting the CFUs. The CFU counts from individual organs were standardized to wet weight (per gram), with each data point corresponding to a single animal and horizontal bars denoting the geometric mean of each experimental group. Kruskal–Wallis (KW) analysis was applied to the spleen bacterial load, and a two-tailed Mann–Whitney U test was applied to the lungs and liver. * *p* < 0.05, and ** *p* < 0.01. (**c**) A histopathology assay. Histological images of hematoxylin and eosin (HE)-stained lung tissues were observed at 48 h after infection with 1 × 10^6^ CFU of SCNJ1 and treated with 50 μL Dep_C, Dep_Y, Dep_Z (at a dose of 20 μg per mouse), or PBS. Mice intranasally administered with PBS served as negative controls. The bacterial clusters in the alveolar cavities are indicated by black arrows. Scale bars, 100 μm.

After intranasal challenge with SCNJ1 (1 × 10^7^ CFU/mouse), 90% of the mice died within 72 h. In contrast, when the mice infected with SCNJ1 were treated with depolymerases (30 μg/mouse) one hour post-infection, their survival rate increased significantly, reaching 100% within 7 days (all, *p* = 0.0003 vs. PBS-treated group). In addition, the survival rates of the safety group and the PBS control group were both 100% within 7 days (Figure 7a).

To further evaluate the therapeutic effect of the depolymerases, after 48 h of infection by intranasal administration of SCNJ1 (1 × 10^6^ CFU/mouse) and being treated with depolymerases (at a dose of 20 μg per mouse) or PBS, the lungs, livers, and spleens of mice were dissected, and the bacterial content in these organs was measured. In the PBS-treated group, the bacterial load in the lungs was approximately 2.2 × 10^11^ CFU per mouse; in the liver, it was approximately 7.3 × 10^8^ CFU per mouse; and in the spleen, it was approximately 7.0 × 10^9^ CFU per mouse. The bacterial loads in the lungs, liver, and spleen of the depolymerase-treated group were significantly decreased (Figure 7b) compared with those of the PBS-treated group (lungs, all, *p* < 0.05; liver, all, *p* < 0.05; spleen, all, *p* < 0.01). These results indicate that all three depolymerases have a significant therapeutic effect on mice infected with the hypervirulent K54 *K. pneumoniae*.

To gain an in-depth understanding of the therapeutic effect of the depolymerases on SCNJ1, 48 h after intranasal infection of SCNJ1 (1 × 10^6^ CFU/mouse), histopathological analysis was performed on the lungs of mice treated with depolymerases (at a dose of 20 μg per mouse) or PBS and the mice of the PBS control group. Compared with the mice that were intranasally administered only with PBS (negative controls), the lung tissues of the mice in the PBS-treated group were filled with a large number of red blood cells, accompanied by obvious exudates, and there were noticeable bacterial clusters in the alveolar cavities (indicated by the arrows in Figure 7c). In the depolymerase-treated groups, the infection also led to the infiltration of inflammatory cells in the lung tissue. However, bacterial aggregates were scarcely observed in the tissue (Figure 7c).

## 4. Discussion

CR-hvKp is commonly recognized as a significant bacterial pathogen that can cause life-threatening diseases in the host [38]. Bacteriophages have the potential to control CR-hvKp infections and are a promising alternative to antibiotics [39]. CR-hvKp is characterized by the high production of CPS, which serves as a receptor for bacteriophages and can be degraded by depolymerases encoded by bacteriophages [40]. In this study, we characterized three depolymerases encoded by three bacteriophages that specifically target the CR-hvKp strain SCNJ1, which has important implications for the development of novel antibacterial agents against CR-hvKp infections [41].

The N-terminal region of the tail fiber protein is assumed to be responsible for the flexible connection to the tail structure or baseplate [42]. Dep_Z shares a conserved domain at its N-terminal region with that of the tail fiber protein of T7 bacteriophages, indicating that Dep_Z may anchor to the phage SCNJ1-Z in a manner analogous to that of T7 bacteriophages. The central region of the amino acid coding sequence of bacteriophage depolymerases commonly functions as the polysaccharide depolymerization site and frequently assumes a right-handed β helical conformation [43]. Pectate lyase can degrade galacturonic acid, which represents a major constituent of bacterial polysaccharides and the plant cell wall [9]. In the central domains of the amino acid coding sequence of Dep_C, Dep_Y, and Dep_Z, there exists a pectin_lyase_fold structure, as found in depolymerases Dpo71 and Depo48, which is assumed to be associated with depolymerase activity [44]. The C-terminal region is crucial for the trimerization of the depolymerase and is considered to be responsible for receptor recognition and binding [45]. The amino acid coding sequences in the C-terminal regions of Dep_C, Dep_Y, and Dep_Z are highly conserved, accounting for their common activity against the CPS of *K. pneumoniae* with serotype K54.

The K54 type is one of the most virulent CPS types of *K. pneumoniae* [46]. Before this study, RaK2gp531 remained the sole documented depolymerase against the K54-type CPS of *K. pneumoniae* by the hydrolytic mechanism [33]. Compared with other highly virulent serotypes of *K. pneumoniae*, the depolymerases that degrade the capsules of serotype K54 remain to be explored. In this study, we demonstrate that depolymerases Dep_C, Dep_Y, and Dep_Z have pronounced specificity for K54 CPS. For RaK2gp531 and the three depolymerases in this work, their pectin lyase regions show a high degree of similarity, which could suggest that their modes of action on CPS are likely to be analogous. Therefore, we speculate that Dep_C, Dep_Y, and Dep_Z, much like RaK2gp531, may also function as hydrolases. As the depolymerases have no killing effect on bacteria, we speculate that they exert their antibacterial effects not through direct bacteriolysis but via the enzymatic degradation of the protective CPS layer. The destruction of CPS leads to the exposure of underlying pathogen-associated molecular patterns (PAMPs), thereby facilitating complement opsonization and subsequent macrophage-mediated phagocytosis [47].

We evaluated the enzymatic activity and stability of Dep_C, Dep_Y, and Dep_Z under different pH and temperature conditions. All three depolymerases retained robust activity even under extreme stresses, consistent with bacteriophage depolymerase properties [48]. Functional assays reveal broad operational ranges spanning 20–50 °C and pH 3.0–12.0, with Dep_C and Dep_Z demonstrating superior catalytic efficiency at low concentrations compared to Dep_Y. The robust stability of depolymerases enhances their therapeutic potential, making them less prone to degradation as drug components and enabling sustained activity under physiological conditions (especially at urine pH 6.5–7.0). This advantage positions them as intriguing candidates for antivirulence agents in a hurdle approach for catheter preservation [28].

Bacterial biofilms are communities of aggregated cells embedded in a self-produced matrix of EPSs [49], which could endow bacteria with increased tolerance to antibiotics and the host immune system compared to growth as planktonic cells [46]. Studies have demonstrated that phage depolymerases can act as adjuvants to facilitate phage infection by disrupting the bacterial biofilm by cleaving the glycosidic bonds of EPSs [50]. In the current study, we show that the recombinant depolymerases Dep_C, Dep_Y, and Dep_Z can efficiently inhibit biofilm formation and degrade mature biofilms of K54-type *K. pneumoniae*, which offers a novel approach for the treatment of biofilm-associated bacterial infections.

It has been known that CPS plays a vital role in bacterial resistance to complement-mediated killing in human serum [37]. As expected, bacteria treated with depolymerases Dep_C, Dep_Y, or Dep_Z become more sensitive to human serum in this study. The treatment of depolymerases can enhance the bacteria’s sensitivity to immune-reactive substances (such as complement) in serum and boost the phagocytosis of bacteria by macrophages by destroying the bacterial CPS layer. However, the depolymerase mixed with serum did not completely eradicate bacteria, and similar observations have been reported in previous studies [51]. Incomplete bacterial eradication might be attributed to the presence of a subgroup of bacteria that are susceptible to depolymerases but resist serum complement-mediated killing [52].

In our work, these three depolymerases demonstrate excellent therapeutic potential in the mouse pneumonia model. The 7-day survival rate of mice infected with *K. pneumoniae* SCNJ1 was significantly improved from 0 to 100% after treatment with Dep_C, Dep_Y, or Dep_Z. The organ burden assays indicate a significantly higher bacterial clearance rate of SCNJ1 in the lungs, liver, and spleen after treatment with depolymerases. The histopathology echoed the bacterial clearance in the lungs of depolymerase-treated mice despite the presence of inflammatory cells. Overall, the therapeutic effect of depolymerases in a mouse model infected with *K. pneumoniae* can be interpreted as a depolymerase-mediated degradation of the CPS, which exposes the bacteria to the mice’s innate immunity. The bacteria are likely to be captured by complement C3 [53], which greatly increases the possibility of them being phagocytosed by macrophages. This ultimately leads to the clearance of bacteria by the host [54]. However, in immunocompromised hosts, the efficacy of depolymerases is likely to be diminished [55]. When monotherapy with depolymerases demonstrates limited therapeutic outcomes, the sequential administration of depolymerases or combination therapy with antibiotics may be warranted.

Phage therapy holds considerable promise for combating drug-resistant pathogens, but its drawbacks should not be ignored, including the risk of virulence gene and resistance gene transmission, as well as the rapid emergence of phage-resistant bacteria, among others [56]. Therefore, some phage-derived enzymes (such as depolymerase) have garnered considerable attention. We admit that resistance to depolymerases will inevitably emerge during treatment. However, the capsule is the sole target of depolymerases, so resistance to depolymerases can only arise from mutations in the capsule. Mutations in bacterial capsules often incur a significant fitness cost, such as increased susceptibility to serum-killing and phagocytosis, and attenuated virulence in the host [34,40]. The bacterial mutant is rapidly cleared by the host immune system, even if it escapes the degrading effect of depolymerase. In contrast, phages generally have multiple target sites, such as pili, lipopolysaccharides, and outer membrane proteins, which enable bacteria to develop resistance through diverse mutational pathways, thereby increasing the frequency of resistant strain emergence. Moreover, some phage-resistant mutants exhibited minimal fitness costs while retaining full virulence [32,57]. Thus, depolymerases are an attractive and promising antivirulence tool for combating drug-resistant pathogens.

In conclusion, we identified three novel depolymerases targeting K54-type hvKp and demonstrated their stability, specificity, and antivirulence efficacy. Future research should concentrate on their biosafety, therapeutic efficacy, and large-scale production before they are applied in clinical practice. Simultaneously, combining synthetic biology and nanotechnology should promote their clinical translation.

## Figures and Tables

**Table 1:** Bacterial strains, plasmids, and phages used in this study.

Strains, Plasmids, or Phages	Descriptions	References or Sources
Strains		
DH5α	*E. coli*, cloning host	Laboratory stock
BL21(DE3)	*E. coli*, expression cell	Laboratory stock
SCNJ1	*K. pneumoniae*, K54 serotype	[16]
NTUH-K2044	*K. pneumoniae*, clinical isolate of K1 serotype	[17]
Plasmid		
pET22b(+)	Vector for depolymerase expression, Amp^R^	Laboratory stock
Phages		
vB_KpnA_SCNJ1-C	*Siphovirus*, targeting *K. pneumoniae* strain SCNJ1	[15]
vB_KpnA_SCNJ1-Y	*Myovirus*, targeting *K. pneumoniae* strain SCNJ1	[15]
vB_KpnA_SCNJ1-Z	*Podovirus*, targeting *K. pneumoniae* strain SCNJ1	[15]

**Table 2:** The host range of Dep_C, Dep_Y, and Dep_Z.

Genus/Species	Bacterial Strain	Capsule Type	Spot Assay		
			Dep_C	Dep_Y	Dep_Z
*Acinetobacter*					
*Acinetobacter baumannii*	ATCC19606		-	-	-
*Enterobacter*					
*Enterobacter cloacae*	SCNJ7		-	-	-
*Escherichia*					
*Escherichia coli*	SCNJ6		-	-	-
*Klebsiella*					
*K. pneumoniae*	SCNJ9	K54	+	+	+
*K. pneumoniae*	SCNJ24	K25	-	-	-
*K. pneumoniae*	SCNJ29	K18	-	-	-
*K. pneumoniae*	SCNJ41	K102	-	-	-
*K. pneumoniae*	SCNJ48	K57	-	-	-
*K. pneumoniae*	SCNJ2	K47	-	-	-
*K. pneumoniae*	SCNJ10	K64	-	-	-

-, no lysis; +, showed halos.

## Data Availability

The source data of this study are deposited in Open Science Framework with the link https://osf.io/rch6z/?view_only=3ab509b52a434a3fa217a541ca682671 (accessed on 10 April 2025).

## Abbreviation

The following abbreviations are used in this manuscript:

CR-hvKp	Carbapenem-resistant hypervirulent *Klebsiella pneumoniae*
CPS	Capsular polysaccharide
SCNJ1-C	vB_KpnA_SCNJ1-C
SCNJ1-Y	vB_KpnS_SCNJ1-Y
SCNJ1-Z	vB_KpnM_SCNJ1-Z
LB	Luria-Bertani
IPTG	Isopropyl-β-D-thiogalactopyranoside
12% SDS-PAGE	12% gradient sodium dodecyl sulfate–polyacrylamide gel electrophoresis
OD600 nm	Optical density at 600 nm
SM buffer	Saline–magnesium buffer
CPC	Cetylpyridinium chloride
EPSs	Extracellular polysaccharides
PAMPs	Pathogen-associated molecular patterns
